# Epidemiological and Molecular Characteristics of HIV-1 Infection in a Sample of Men Who Have Sex With Men in Brazil: Phylogeography of Major Subtype B and F1 Transmission Clusters

**DOI:** 10.3389/fmicb.2020.589937

**Published:** 2020-11-27

**Authors:** Ágabo Macêdo da Costa e Silva, Mônica Nogueira da Guarda Reis, Thaís Augusto Marinho, Nara Rúbia de Freitas, Sheila Araújo Teles, Márcia Alves Dias de Matos, Megmar Aparecida dos Santos Carneiro, Gonzalo Bello, Mariane Martins Araújo Stefani, Regina Maria Bringel Martins

**Affiliations:** ^1^Institute of Tropical Pathology and Public Health, Federal University of Goiás, Goiânia, Brazil; ^2^Faculty of Nursing, Federal University of Goiás, Goiânia, Brazil; ^3^AIDS and Molecular Immunology Laboratory, Oswaldo Cruz Institute, Oswaldo Cruz Foundation, Rio de Janeiro, Brazil

**Keywords:** HIV-1, epidemiology, genetic diversity, transmitted drug resistance, MSM

## Abstract

This study describes human immunodeficiency virus 1 (HIV-1) prevalence, associated factors, viral genetic diversity, transmitted drug resistance (TDR), and acquired drug resistance mutations (DRM) among a population of 522 men who have sex with men (MSM) recruited by the respondent-driven sampling (RDS) method, in Goiânia city, the capital of the State of Goiás, Central-Western Brazil. All serum samples were tested using a four-generation enzyme-linked immunosorbent assay (ELISA), and reactive samples were confirmed by immunoblotting. Plasma RNA or proviral DNA was extracted, and partial *polymerase* (*pol*) gene including the protease/reverse transcriptase (PR/RT) region was amplified and sequenced. HIV-1 subtypes were identified by phylogenetic inference and by bootscan analysis. The time and location of the ancestral strains that originated the transmission clusters were estimated by a Bayesian phylogeographic approach. TDR and DRM were identified using the Stanford databases. Overall, HIV-1 prevalence was 17.6% (95% CI: 12.6–23.5). Self-declared black skin color, receptive anal intercourse, sex with drug user partner, and history of sexually transmitted infections were factors associated with HIV-1 infection. Of 105 HIV-1-positive samples, 78 (74.3%) were sequenced and subtyped as B (65.4%), F1 (20.5%), C (3.8%), and BF1 (10.3%). Most HIV-1 subtype B sequences (67%; 34 out of 51) branched within 12 monophyletic clusters of variable sizes, which probably arose in the State of Goiás between the 1980s and 2010s. Most subtype F1 sequences (*n* = 14, 88%) branched in a single monophyletic cluster that probably arose in Goiás around the late 1990s. Among 78 samples sequenced, three were from patients under antiretroviral therapy (ART); two presented DRM. Among 75 ART-naïve patients, TDR was identified in 13 (17.3%; CI 95%: 9.6–27.8). Resistance mutations to non-nucleoside reverse transcriptase inhibitors (NNRTI) predominated (14.7%), followed by nucleoside reverse transcriptase inhibitor (NRTI) mutations (5.3%) and protease inhibitor (PI) mutations (1.3%). This study shows a high prevalence of HIV-1 associated with sexual risk behaviors, high rate of TDR, and high genetic diversity driven by the local expansion of different subtype B and F1 strains. These findings can contribute to the understanding about the dissemination and epidemiological and molecular characteristics of HIV-1 among the population of MSM living away from the epicenter of epidemics in Brazil.

## Introduction

Human immunodeficiency virus 1 (HIV-1) infection continues to be a major global public health challenge (UNAIDS, 2019; WHO, 2019). One of the hallmarks of HIV-1 is its extensive genetic diversity and rapid evolution within and between infected individuals, and regardless of the development of strong immune responses, its diversity contributes to both viral persistence and the selection of antiretroviral (ARV) resistance, representing a challenge for the development of vaccines and curative therapies (Van Zyl et al., 2018; Tough and McLaren, 2019). Globally, the molecular epidemiology of HIV-1 is complex and dynamic with the predominance of subtype C which corresponds to more than 50%, followed by subtypes B and A in addition to other subtypes and circulating recombinant forms (CRFs) (Bbosa et al., 2019).

HIV-1 infection worldwide continues to grow among men who have sex with men (MSM) (Beyrer et al., 2016; Chapin-Bardales et al., 2018). Estimates indicate that MSM are almost 22 times more likely to be infected with HIV-1 compared to the general population (UNAIDS, 2019). The epidemic is reemerging among MSM as a serious public health problem even in high-income countries (Chapin-Bardales et al., 2018). Sexual behavior factors as high rate of unprotected anal sex and multiple sexual partners are known to increase the vulnerability to infection (Pines et al., 2016; Rocha et al., 2020). Also, social and structural factors, such as stigma, discrimination, and lack of or poor access to prevention programs, probably contribute to the high burden of HIV-1 infection among MSM (Chakrapani et al., 2019).

Brazil was the first developing country in the world to offer universal free antiretroviral therapy (ART) by the governmental public health system. Since then, acquired immunodeficiency syndrome (AIDS) incidence and AIDS-associated mortality rates have decreased in the epicenter of the epidemic and in most Brazilian states (Brasil. Ministério da Saúde. Secretaria de Vigilância em Saúde. Departamento de Vigilância Prevenção e Controle das Ist, do HIV/AIDS e das Hepatites Virais – DIAHV, 2019). However, there have been concerns about the emergence and transmission of HIV-1, especially in specific populations such as MSM (Guimarães et al., 2018; Kerr et al., 2018; Rocha et al., 2020). A recent multicentric study conducted among MSM in 12 Brazilian State capitals located in the five geographic Brazilian regions has reported an overall HIV-1 prevalence of 18.4%, ranging from 5.8% in Brasília to 24.8% in São Paulo (Kerr et al., 2018). A previous Brazilian multicentric study showed that the highest HIV prevalence (51.9%) was detected in a group of injecting drug users MSM (gays and bisexuals) (Ferreira et al., 2006). However, data on HIV-1 genetic diversity and drug resistance mutations among MSM populations in Brazil are still scarce (Bermúdez-Aza et al., 2011; Tupinambás et al., 2013).

This study aimed to estimate HIV-1 prevalence and associated factors among a population of MSM from Central-Western Brazil. HIV-1 genetic diversity and the presence of mutations associated with drug resistance were also described. Additionally, phylogenetic and phylogeographic analysis of sequences linked with epidemiological data indicated HIV-1 dissemination trends in this population.

## Materials and Methods

### Study Population

This cross-sectional study was conducted among MSM in Goiânia city (1.5 million inhabitants), the capital of the State of Goiás, located in Central-Western Brazil, from March to November 2014. Participants were recruited using the respondent-driven sampling (RDS) method, a chain-referral sampling strategy based on social networks of the target population, which is considered an efficient data collection method to study hidden populations (Heckathorn, 1997). In this study, as previously described (Oliveira et al., 2016), the recruitment process started with a non-random selection of five key members of the MSM population denominated as “seeds” who received three numbered referral coupons to recruit members of their social network. These recruits also received the same number of referral coupons to recruit additional participants. This process was repeated until the desired sample size was achieved. The sample size calculation considered the expected design effect of 2.0 (Salganik, 2006), a precision of 4.4%, and an estimated prevalence for HIV infection of 14.2% (Kerr et al., 2013) with confidence interval of 95%, resulting in a minimum sample size of 484 participants. The eligibility criteria for the study were being born male, reporting to have had sex with another man in the last 12 months, aged 18 years or older, living in Goiânia, and presenting a valid recruitment coupon. All participants were previously informed about the aims of the study and signed the informed consent form. Participants were interviewed using a standardized questionnaire to collect data on sociodemographic and risk behaviors for HIV-1 infection. Then, a sample of venous blood (10 mL) was collected from each participant for laboratory tests. The protocol used for this study was approved by the Ethics Committee of the Federal University of Goiás (reference number 497374).

### Serological Tests

All serum samples were tested using a four-generation enzyme-linked immunosorbent assay (ELISA) for the simultaneous detection of HIV-1 p24 antigen and anti-HIV-1/2 antibodies (HIV Ag/Ab ELISA 4^a^ Generation test, Wiener Lab, Rosario, Argentina). The reactive samples were confirmed for the presence of HIV-1 antibodies by immunoblotting (New Lav Blot I, Bio-Rad, Marnes-la-Coquette, France).

### Amplification and Sequencing of HIV-1 *Polymerase* (*pol*) Gene

RNA was extracted from all plasma samples of ELISA seropositive participants using the QIAamp^®^ Viral RNA Mini Kit (Qiagen, Hilden, Germany), followed by reverse transcription (Life Technologies, Carlsbad, CA, United States), and cDNA was used as the target for a nested polymerase chain reaction (PCR) of the *pol* gene. The entire protease (PR) region (849 bp) and approximately 750 bp of the reverse transcriptase (RT) fragment were amplified as described previously (Cardoso et al., 2009). Non-amplified samples were subjected to genomic DNA extraction from whole blood using the QIAamp^®^ DNA Blood Mini Kit (Qiagen, Hilden, Germany). After amplicon purification (QIAquick^®^ PCR Purification Kit, Qiagen, Hilden, Germany), genomic sequencing was performed (BigDye Terminator Sequencing Kit v. 3.1, Applied Biosystems, Foster City, CA, United States; ABI Prism 3130xl Genetic Analyzer, Applied Biosystems, Foster City, CA, United States). All HIV-1 sequences generated in this study were deposited in the GenBank database (accession numbers MN509088–MN509165).

### HIV-1 Genetic Diversity Analysis

The HIV-1 subtypes were identified using the REGA HIV-1 Subtyping Tool version 3.0 (Oliveira et al., 2005) and by phylogenetic inference using reference sequences obtained from the GenBank and the Los Alamos HIV Databases, including HIV-1 group M subtypes (A-D, F1-H, J, K) and CRF-BF1 sequences with recombination in the *pol*^1^. Study sequences were aligned using the Clustal X 2.0 implemented by the BioEdit 7.2.0 program (Hall, 1999). Phylogenetic trees were generated using the neighbor-joining (NJ) method under Kimura’s two-parameter substitution model using Molecular Evolutionary Genetics Analysis (MEGA) software version 6.0 (Tamura et al., 2013), and bootstrap values (1,000 replicates) above 70% were considered significant. The Simplot version 3.5.1 software was used for recombination analyses (200-bp sliding window, 20-bp step size increments, NJ method under Kimura’s two-parameter correction with 1,000 bootstrap replicates) (Lole et al., 1999). Informative site analyses were used to characterize recombination breakpoints compared with consensus sequences from Brazilian HIV-1 subtypes B and F generated in the DAMBE program (Xia and Xie, 2001). Each sequence fragment assigned to a specific HIV-1 subtype was confirmed by separate NJ phylogenetic analysis as described above.

### Maximum Likelihood (ML) Phylogenetic Analysis

HIV-1 subtypes B and F1 sequences from MSM individuals from Goiânia were aligned with other HIV-1 Brazilian sequences of the same subtypes sampled at different states that were available at the Los Alamos HIV Sequence Database. ML phylogenetic trees were reconstructed with the PhyML program (Guindon et al., 2010) using an online web server (Guindon et al., 2005) under the best-fit nucleotide substitution model selected with the SMS tool (Lefort et al., 2017), the SPR branch-swapping algorithm of heuristic tree search, and the approximate likelihood-ratio test (aLRT) (Anisimova and Gascuel, 2006) of reliability tree topology. The ML trees were visualized using the FigTree v1.4.4 program (Rambaut, 2009).

### Bayesian Phylogeographic Analysis

To reconstruct the most probable source location and the time of origin of major subtypes B and F1 MSM clusters here identified in Goiânia, we selected a subset of Brazilian subtypes B (*n* = 54) and F1 sequences (*n* = 18) that branched with high support (a*LRT* = 0.90) with subtype B and F1 MSM sequences from Goiânia in the ML phylogenetic trees. The evolutionary rate, time of the most recent common ancestor (T_*MRCA*_; years), and spatial diffusion pattern were jointly estimated using the Bayesian Markov Chain Monte Carlo (MCMC) approach as implemented in BEAST v1.10 (Drummond et al., 2002; Drummond and Rambaut, 2007) with BEAGLE to improve run time (Suchard and Rambaut, 2009). Because regression analysis using TempEst program (Rambaut et al., 2016) revealed that the HIV-1 *pol* dataset here compiled does not contain sufficient temporal signal for reliable time-scale estimations (data not shown), the time-scale was reconstructed using a uniform prior distribution on the substitution rate that encompasses mean values previously estimated for the HIV-1 *pol* gene (1.5–2.5 × 10^–3^ subst./site/year) (Hue et al., 2005). Bayesian MCMC analyses were performed using the GTR + I + G nucleotide substitution model (Tavaré, 1986), a Bayesian Skyline coalescent tree prior (Drummond et al., 2005), a relaxed uncorrelated lognormal molecular clock model (Drummond et al., 2006), and a reversible discrete phylogeography model (Lemey et al., 2009) with a continuous-time Markov chain (CTMC) rate reference prior (Ferreira and Ma, 2008). MCMC chains were run for 10 × 10^6^ generations, and convergence (effective sample size > 200) and uncertainty [95% highest probability density (HPD) values] in parameter estimates were assessed using the TRACER v1.6 program (Rambaut and Drummond, 2007). The maximum clade credibility (MCC) trees were summarized with TreeAnnotator v1.10 and visualized with FigTree v1.4.4.

### HIV-1 Drug Resistance Mutations

Drug resistance mutations (DRM) and susceptibility profiles were analyzed employing the Stanford HIV Drug Resistance Database v. 8.8^2^, which indicate HIV mutations associated with transmitted and acquired resistance to the most commonly used antiretroviral drugs. The rate of transmitted drug resistance (TDR) among ART-naïve participants was analyzed using the Calibrated Population Resistance (CPR) tool employing the Stanford Surveillance Drug Resistance Mutation (SDRM) database (Gifford et al., 2009).

### Data Analysis

Data were analyzed using the RDS Analysis Tool (RDSAT), version 7.1.46^3^ in order to provide weights controlling for selective recruitment bias and social network size (Heckathorn, 1997). Adjusted frequency distributions were calculated with 95% confidence intervals (95% CI). The representation of the recruitment network with the distribution of the HIV-1 infection cases was performed using NetDraw software^4^ (Borgatti, 2002). The population weights generated by RDSAT were transferred to the STATA software, version 15.0, for analysis of factors associated with HIV-1 infection. Bivariate and multiple analyses were conducted using complex sample routines employing the “survey” package. The bivariate logistic regression indicated associations between the dependent variable and each independent variable. Then, variables with *p*-value < 0.05 were included in a multiple logistic regression model to adjust for confounding variables. The results of the multivariate regression model were presented as adjusted odds ratio (ORadj) with 95% CI. The statistical significance of the analyses was established by Wald’s chi-square. The Hosmer and Lemeshow test used to verify the quality of the fit of the regression model showed a *p*-value of 0.699, which indicates good quality of fit (Hosmer et al., 1997). A *p*-value less than 0.05 was considered as statistically significant throughout analyses.

## Results

Based on the respondent-driven sampling method, a total of 522 MSM was included in this study: five were seeds and 517 recruits. As shown in Table 1, most participants were young (≤25 years old, 60%), self-identified as gay (74.9%), have brown or mixed self-reported skin color (*pardo*, 59%), single (76.9%), had attended high school (10–12 years of formal education, 63.9%), and were in the lowest economic class in Brazil (E, 61.3%). A minority of MSM reported previous blood transfusion (5.3%), acupuncture treatment (2.9%), dental treatment with a non-graduated professional (4.3%), or a history of incarceration (6.7%). Almost half of the participants had tattooing and/or piercings (42.3%), and a quarter reported illicit drugs use (25.1%). Regarding sexual behaviors, the majority of MSM (59.7%) reported more than 10 lifetime sexual partners, receptive anal intercourse (75.1%), sex with women (60.2%), and alcohol consumption during sex (61.5%). Almost half of the individuals reported other high-risk practices such as unprotected anal intercourse (44.3%) and having sex with drug users (42.2%; mostly sex with non-injecting illicit drug users). Approximately one third of the study population reported group sex (35.1%), sex for money (33.1%), rectal trauma with bleeding (34.2%), and history of sexually transmitted infections (STI; 30.5%). Some MSM reported sex against will (18.3%) and sex with STI partner (11.9%). Additionally, 19.8 and 15.4% were positive for syphilis and hepatitis B virus (HBV: hepatitis B surface antigen, HBsAg, and/or antibodies against hepatitis B core antigen, anti-HBc markers), respectively.

**TABLE 1:** Characteristics of the studied MSM from Goiânia, Central-Western Brazil.

**Variables**	***n***	**Unweighted %**	**Weighted by RDSAT^*a*^**	
			**%**	**95% CI**
**Age (years)**				
≤25	338	64.8	60.0	50.8–68.5
>25	184	35.22	40.0	31.5–49.2
**Self-identification**				
Bisexual	76	14.6	19.4	13.4–27.2
Gay	355	68.0	74.9	67.2–81.4
Transvestite	91	17.4	5.7	3.8–8.3
**Skin color/race (self-reported)**				
White	103	19.7	18.9	13.5–25.7
Brown (*pardo*)	296	56.7	59.0	50.0–67.4
Black	91	17.4	16.6	9.9–26.4
Others (Indigenous or Asian)	32	6.1	5.5	2.8–9.1
**Marital status**				
Single	420	80.5	76.9	66.9–84.5
Married/stable relationship	94	18.0	20.1	12.9–9.9
Separated/divorced	8	1.5	3.1	0.9–9.4
**Education (years)^*b*^**				
≥13	136	26.2	22.4	17.0–28.9
10–12	307	59.0	63.9	55.9–71.3
≤9	77	14.8	13.7	9.0–20.3
**Economic class^*c*^**				
A/B	7	1.3	0.4	0.1–1.7
C	81	15.5	9.9	6.6–14.5
D	140	26.8	28.4	52.1–69.7
E	294	56.3	61.3	52.1–69.7
**Blood transfusion lifetime^*b*^**				
No	491	95.2	94.7	89.4–97.5
Yes	25	4.8	5.3	2.5–10.6
**Acupuncture^*b*^**				
No	492	95.7	97.1	94.3–98.6
Yes	22	4.3	2.9	1.4–5.7
**Tattoo/piercing^*b*^**				
No	223	43.4	57.7	49.1–65.8
Yes	291	56.6	42.3	34.2–50.9
**Ever used any illicit drug^*b*^**				
No	280	53.6	74.9	68.3–80.5
Yes	241	46.2	25.1	19.5–31.7
**Dental treatment with ungraduated professional^*b*^**				
No	477	94.5	95.7	91.8–97.7
Yes	28	5.5	4.3	2.2–8.0
**Incarceration^*b*^**				
No	469	90.4	93.3	87.5–96.6
Yes	50	9.6	6.7	3.4–12.5
**Number of lifetime sexual partners^*b*^**				
≤10	156	29.9	40.3	51.9–65.4
>10	365	70.1	59.7	51.2–67.7
**Receptive anal intercourse^*b*^**				
No	88	16.9	24.9	17.8–33.6
Yes	433	83.1	75.1	66.4–82.2
**Condom use at anal intercourse^*b*^**				
Always	252	48.6	55.7	47.1–64.0
Not always	267	51.4	44.3	36.0–52.9
**Sex with women^*b*^**				
No	232	44.5	39.8	32.1–48.1
Yes	289	55.5	60.2	51,9–67.9
**Group sex^*b*^**				
No	240	46.2	64.9	57.0–72.0
Yes	279	53.8	35.1	28.0–43.0
**Sex against will^*b*^**				
No	374	72.1	81.7	75.7–86.5
Yes	145	27.9	18.3	13.5–24.3
**Sex for money^*b*^**				
No	281	54.0	66.9	57.4–75.3
Yes	239	46.0	33.1	24.7–42.6
**Ever used alcohol during sex^*b*^**				
No	165	31.7	38.5	30.6–47.1
Yes	356	68.3	61.5	52.9–69.4
**Sex with STI partner**				
No	458	87.7	88.1	83.4–93.2
Yes	64	12.3	11.9	6.8–16.6
**Sex with drug user partner^*b*^**				
No	199	39.0	57.8	49.3–65.8
Yes	311	61.0	42.2	34.2–50.7
**Sex with bleeding^*b*^**				
No	283	59.1	65.8	57.2–73.5
Yes	196	40.9	34.2	26,5–42.8
**STI^*b*^**				
No	388	75.2	69.5	59.1–78.3
Yes	128	24.8	30.5	21.7–40.9
**Syphilis status**				
Negative	413	79.1	80.2	74.6–85.5
Positive	109	20	19.8	14.5–25.5
**HBV status^*d*^**				
Negative	445	85.2	84.6	81.8–90.7
Positive	77	14.8	15.4	8.7–25.8

*MSM, men who have sex with men; RDSAT, RDS analysis tool; CI, confidence interval; STI, sexually transmitted infections; HBV, hepatitis B virus. ^*a*^Population-based estimates are based on analysis in RDSAT, which is adjusted for personal network sizes and recruitment patterns. ^*b*^Missing data are not shown. ^*c*^Brazilian government criteria to classify individuals into five economic classes (A, the highest, to E, the lowest), which were based on the total household income (Brasil. Secretaria de Assuntos Estratégicos da Presidência da República do Brasil, 2014): A/B (≥US$ 3,725), C (US$ 865–3,724), D (US$ 541–864), and E (<US$ 541; US$ 1.00 was approximately equal to R$ 2.32 in March 2014). ^*d*^HBsAg, hepatitis B surface antigen and/or anti-HBc, antibodies against hepatitis B core antigen.*

**FIGURE 1:**
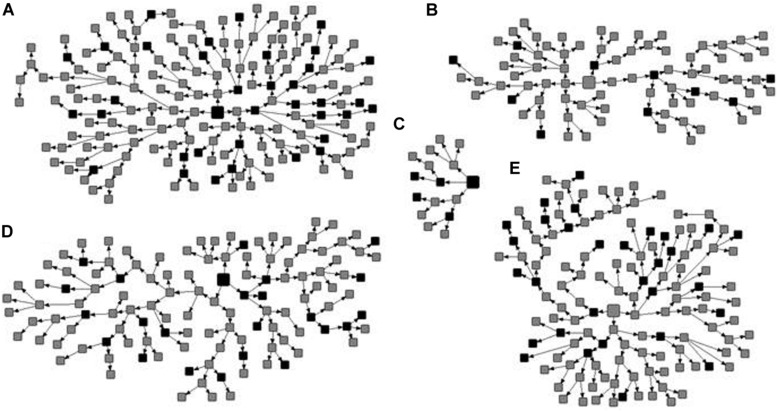
Recruitment networks **(A–E)** of 522 men who have sex with men (MSM) from Central-Western Brazil. The seeds are indicated by large squares and recruits by small squares. The identified HIV-1 seropositive MSM are shown in black and the other participants in gray.

Among the 522 MSM enrolled, 105 were HIV-1 seropositive, resulting in a prevalence of 17.6% (95% CI: 12.6–23.5) adjusted by RDSAT. Figure 1 shows the representation of the five recruitment networks (A–E), highlighting the identified HIV-1-positive MSM. Bivariate analysis results revealed the following demographic and behavioral factors as significantly associated with HIV-1 infection among MSM (Table 2): age over 25 years, self-declared black skin color, reported dental treatment with ungraduated professional, receptive anal intercourse, group sex, sex for money, sex with STI partner, sex with drug user partner, history of STI, and HBV-positive status. These variables were included in a multivariate analysis using a logistic regression model. As shown in Table 3, in our study population self-declared black skin color (*p* = 0.008), receptive anal intercourse (*p* = 0.046), sex with drug user partner (*p* = 0.045), and history of STI (*p* = 0.006) were independently associated with HIV-1 infection.

**TABLE 2:** Demographic and behavioral characteristics associated with HIV infection among MSM from Goiânia, Central-Western Brazil.

**Variables**	**HIV Pos./total**	**Unweighted %**	**Weighted^*a*^ % (95% CI)**	**OR (95% CI)**	***p*-value**
**Age (years)**					
≤25	55/338	16.3	13.3 (7.9–19.4)	1.00	
>25	50/184	27.2	24.6 (15.2–35.4)	1.92 (1.24–2.97)	0.003
**Self-identification**					
Bisexual	9/76	11.8	12.5 (2.8–22.9)	1.00	
Gay	76/355	21.4	19.3 (13.4–25.6)	2.03 (0.97–4.25)	0.061
Transvestite	20/91	22.0	15.1 (2.0–29.4)	2.09 (0.89–4.93)	0.089
**Skin color/race (self-reported)**					
White	15/103	14.6	14.0 (4.9–26.3)	1.00	
Brown (*pardo*)	61/296	20.6	19.9 (12.3–28.0)	1.52 (0.82–2.82)	0.181
Black	25/91	27.5	16.7 (8.6–27.6)	2.22 (1.09–4.54)	0.029
Others (Indigenous or Asian)	4/32	12.5	15.1 (0.0–44.6)	0.83 (0.26–2.73)	0.770
**Marital status**					
Single	84/420	20.0	16.6 (10.9–23.0)	1.00	
Married/stable relationship	19/94	20.2	19.8 (8.4–33.0)	1.01 (0.58–1.77)	0.963
Separated/divorced	2/8	25.0	50.5 (0.0–96.9)	1.33 (0.26–6.72)	0.727
**Education (years)^*b*^**					
≥13	26/136	19.2	16.6 (7.3–27.3)	1.00	
10–12	62/307	20.2	18.0 (11.8–25.8)	1.07 (0.64–1.79)	0.793
≤9	17/77	22.1	20.7 (4.3–37.0)	1.20 (0.60–2.39)	0.606
**Economic class^*c*^**					
E	56/294	19.0	16.9 (9.7–23.0)	1.00	
D	30/140	21.4	17.4 (6.8–28.0)	1.16 (0.71–1.91)	0.561
C	19/81	23.5	26.7 (13.3–46.1)	1.30 (0.72–2.35)	0.381
A/B	0/7	0.0	0.0 (0.0–0.0)	–	–
**Blood transfusion lifetime^*b*^**					
No	98/491	20.0	17.6 (12.1–23.3)	1.00	
Yes	5/25	20.0	29.3 (3.1–60.0)	1.00 (0.37–2.75)	0.996
**Acupuncture^*b*^**					
No	97/492	19.7	18.2 (13.1–24.9)	1.00	
Yes	7/22	31.8	20.1 (3.1–50.3)	1.90 (0.75–4.79)	0.173
**Tattoo/piercing^*b*^**					
No	53/223	23.8	20.5 (12.6–29.4)	1.00	
Yes	51/291	17.5	14.8 (8.8–22.5)	0.68 (0.44–1.05)	0.082
**Ever used any illicit drug^*b*^**					
No	57/280	20.4	18.5 (11.7–25.4)	1.00	
Yes	48/241	19.9	16.3 (9.4–24.6)	0.97 (0.63–1.50)	0.901
**Dental treatment with ungraduated professional**					
No	93/477	19.5	17.4 (12.6–23.8)	1.00	
Yes	10/28	35.7	36.0 (2.9–66.7)	2.29 (1.02–5.13)	0.043
**Incarceration^*b*^**					
No	89/469	19.0	18.0 (12.4–23.8)	1.00	
Yes	15/50	30.0	16.8 (3.8–42.0)	1.83 (0.96–3.50)	0.067
**Number of lifetime sexual partners^*b*^**					
≤10	27/156	17.3	14.7 (8.0–22.9)	1.00	
>10	78/365	21.4	20.3 (12.8–28.1)	1.30 (0.80–2.11)	0.290
**Receptive anal intercourse^*b*^**					
No	7/88	8.0	9.0 (0.3–21.0)	1.00	
Yes	98/433	22.6	19.8 (14.2–26.6)	3.38 (1.51–7.57)	0.002
**Condom use at anal intercourse^*b*^**					
Always	49/252	19.4	11.3 (6.7–16.9)	1.00	
Not always	56/267	21.0	25.7 (16.3–35.6)	1.10 (0.82–1.69)	0.665
**Sex with women^*b*^**					
No	51/232	22.0	18.7 (11.0–27.2)	1.00	
Yes	54/289	18.7	17.0 (10.9–25.1)	0.81 (0.53–1.25)	0.351
**Group sex^*b*^**					
No	37/240	15.4	17.1 (9.7–23.9)	1.00	
Yes	68/279	24.4	18.8 (11.2–27.3)	1.77 (1.13–2.76)	0.012
**Sex against will^*b*^**					
No	74/374	19.8	15.5 (10.1–21.1)	1.00	
Yes	31/145	21.4	27.5 (13.0–41.3)	1.10 (0.69–1.77)	0.685
**Sex for money^*b*^**					
No	45/281	16.0	14.6 (9.0–21.0)	1.00	
Yes	60/239	25.1	25.8 (14.5–36.4)	1.76 (1.14–2.71)	0.011
**Ever used alcohol during sex^*b*^**					
No	34/165	20.6	18.6 (10.5–27.7)	1.00	
Yes	71/356	19.9	17.6 (10.9–24.6)	0.96 (0.61–1.52)	0.861
**Sex with STI partner**					
No	83/458	18.1	14.9 (9.8–20.7)	1.00	
Yes	22/64	34.4	38.9 (20.2–62.9)	2.37 (1.34–4.18)	0.003
**Sex with drug user partner^*b*^**					
No	27/199	13.6	12.3 (6.5–19.1)	1.00	
Yes	78/311	25.1	26.7 (17.9–36.6)	2.13 (1.32–3.45)	0.002
**Sex with bleeding^*b*^**					
No	52/283	18.4	13.6 (8.5–19.6)	1.00	
Yes	44/196	22.5	25.6 (12.4–36.7)	1.29 (0.82–2.02)	0.274
**STI^*b*^**					
No	61/388	15.7	14.2 (8.8–20.6)	1.00	
Yes	43/128	33.6	27.7 (13.6–38.0)	2.71 (1.72–4.28)	<0.001
**Syphilis status**					
Negative	79/413	19.1	17.2 (11.2–23.4)	1.00	
Positive	26/109	24.0	19.7 (8.2–32.9)	1.32 (0.80–2.20)	0.276
**HBV status^*d*^**					
Negative	82/445	18.4	13.5 (8.8–18.9)	1.00	
Positive	23/77	30.0	45.9 (25.5–62.9)	1.89 (1.09–3.25)	0.022

*HIV, human immunodeficiency virus; MSM, men who have sex with men; CI, confidence interval; OR, odds ratio; STI, sexually transmitted infections; HBV, hepatitis B virus. ^*a*^Population-based estimates are based on analysis in RDSAT, which is adjusted for personal network sizes and recruitment patterns. ^*b*^Missing data are not shown. ^*c*^Brazilian government criteria to classify individuals into five economic classes (A, the highest, to E, the lowest), which were based on the total household income (Brasil. Secretaria de Assuntos Estratégicos da Presidência da República do Brasil, 2014): A/B (≥US$ 3,725), C (US$ 865–3,724), D (US$ 541-864), and E (<US$ 541; US$ 1.00 was approximately equal to R$ 2.32 in March 2014). ^*d*^HBsAg, hepatitis B surface antigen and/or anti-HBc, antibodies against hepatitis B core antigen.*

**TABLE 3:** Multivariate analysis of factors associated with HIV-1 infection among MSM from Goiânia, Central-Western Brazil.

**Variables**	**Adjusted OR (95% CI)^*a*^**	***p*-value**
**Age (years)**		
≤25	1.00	
>25	1.17 (0.67–2.04)	0.585
**Skin color/race (self-reported)**		
White	1.00	
Brown (*pardo*)	1.82 (0.91–3.62)	0.089
Black	2.92 (1.32–6.44)	0.008
Others (Indigenous or Asian)	0.95 (0.28–3.27)	0.936
**Dental treatment with ungraduated professional**		
No	1.00	
Yes	1.82 (0.76–4.34)	0.177
**Receptive anal intercourse**		
No	1.00	
Yes	2.42 (1.02–5.78)	0.046
**Group sex**		
No	1.00	
Yes	1.02 (0.60–1.73)	0.938
**Sex for money**		
No	1.00	
Yes	1.35 (0.82–2.25)	0.242
**Sex with STI partner**		
No	1.00	
Yes	1.68 (0.90–3.13)	0.105
**Sex with drug user partner**		
No	1.00	
Yes	1.77 (1.01–3.08)	0.045
**STI**		
No	1.00	
Yes	2.07 (1.23–3.49)	0.006
**HBV status**^*b*^		
Negative	1.00	
Positive	0.39 (0.09–1.68)	0.206

**HIV, human immunodeficiency virus; MSM, men who have sex with men; OR, odds ratio; CI, confidence interval; STI, sexually transmitted infections; HBV, hepatitis B virus. ^*a*^Adjusted odds ratio for the following variables: age, skin color/race, dental treatment with ungraduated professional, receptive anal intercourse, group sex, sex for money, sex with STI partner, sex with drug user partner, STI and HBV status. ^*b*^HBsAg, hepatitis B surface antigen and/or anti-HBc, antibodies against hepatitis B core antigen.**

**FIGURE 2:**
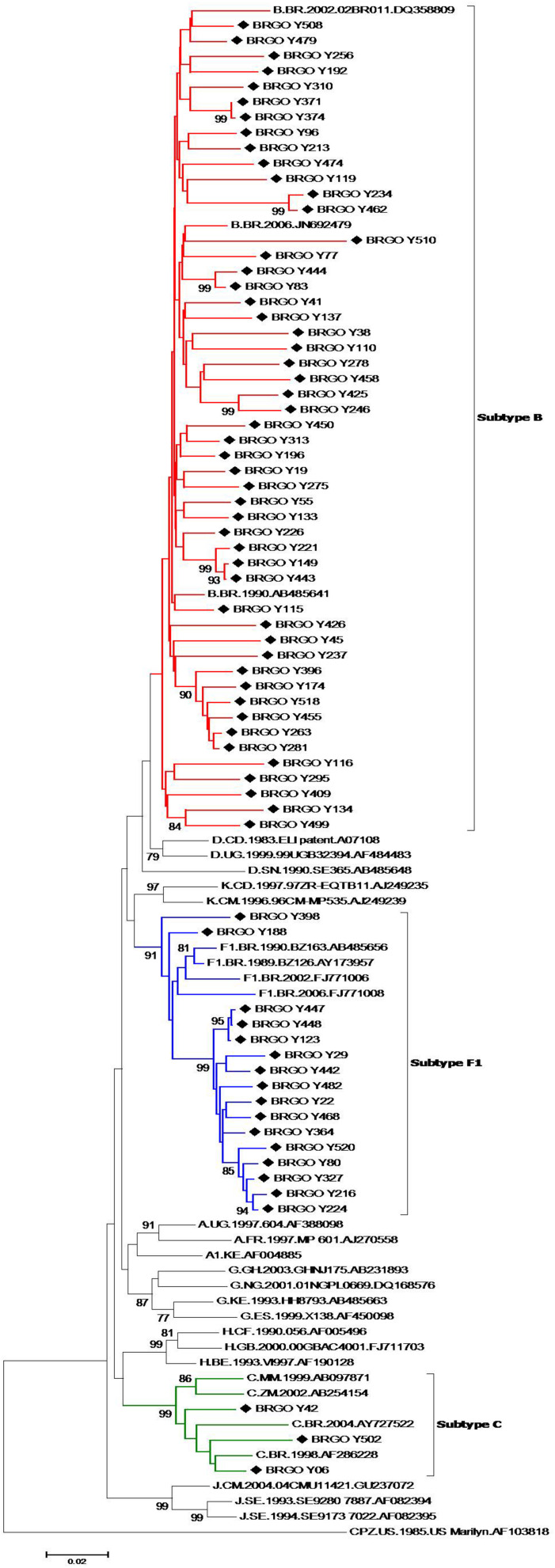
Phylogenetic tree analyses of HIV in protease (PR) and reverse transcriptase (RT) fragments among men who have sex with men (MSM) from Central-Western Brazil. In the mosaic structure, the red color stands for HIV-1 subtype B, blue color stands for subtype F1, and green color stands for subtype C. The sequences described in our study are distinguished from the sequences retrieved from the GenBank by a diamond signal (◆). Phylogenetic trees were generated using the neighbor-joining (NJ) method under Kimura’s two-parameter substitution model using Molecular Evolutionary Genetics Analysis (MEGA) software version 6.0, and bootstrap values (1,000 replicates) above 70% were considered significant. Thirty reference sequences obtained from GenBank were used in the comparative phylogenetic analysis, including 29 HIV-1 group M subtypes (A–D, F1–H, J, K) and one simian immunodeficiency virus sequence from chimpanzee (SIVcpz), used as the out group.

**FIGURE 3:**
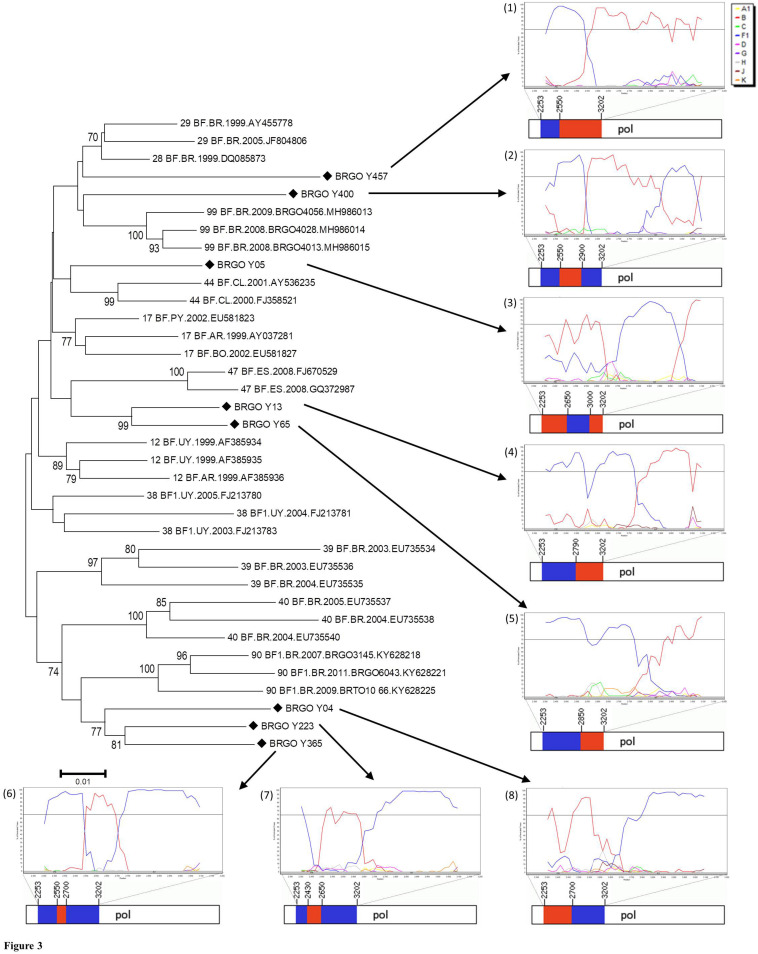
Phylogenetic analyses of eight HIV-1 *pol* sequences of BF1 recombinants among men who have sex with men (MSM) from Central-Western Brazil. All CRF_BF depicting recombination breakpoints in *pol* gene were included in the analysis. The sequences described in our study are distinguished from the sequences retrieved from the Los Alamos HIV Database by a diamond signal (◆). The NJ method and Kimura two-parameter evolutionary model with 1,000 replicates bootstrap values were applied. The numbers from (1) to (8) represent the Simplot Bootscan analysis and the mosaic structures containing the breakpoint positions of each recombinant. Bootscan analysis was performed in a 200-nt sliding window advanced in 20-nt step size increments (1,000 bootstrap replicates), where the red color stands for subtype B, blue stands for subtype F1. In the mosaic structure representations of BF1 isolates, the breakpoint positions are indicated according numeration to HIV-1 genome position; the red color stands for subtype B, and blue stands for subtype F1.

Of 105 HIV-1-positive MSM identified, 79 (75.2%) were HIV-1 RNA and/or DNA positive and 78 (74.3%) had PR and RT regions of the HIV-1 *pol* gene sequenced and analyzed. Most HIV-1 *pol* sequences analyzed (89.7%, 70 out of 78) clustered with a single HIV-1 subtype in both PR and RT regions: 51 subtype B (65.4%), 16 subtype F1 (20.5%), and three subtype C (3.8%) (Figure 2). Moreover, eight MSM (10.3%) had BF1 recombinant isolates with different breakpoint positions: F1^*PR*^/F1B^*RT*^ (*n* = 2); F1^*PR*^/BF1^*RT*^ (*n* = 2), B^*PR*^/BF1^*RT*^ (*n* = 1), B^*PR*^/BF1B^*RT*^ (*n* = 1), F1^*PR*^/B^*RT*^ (*n* = 1), and F1B^*PR*^/BF1^*RT*^ (*n* = 1) (Figure 3).

The HIV-1 subtype B (*n* = 51) and F1 (*n* = 16) sequences from MSM individuals from Goiânia were aligned with subtype B (*n* = 2,010) and F1 (*n* = 299) Brazilian sequences with information about sampling state that were available at the Los Alamos HIV Sequence Database. The ML analysis revealed that a significant proportion of the HIV-1 subtype B sequences from Goiânia here recovered (*n* = 34, 67%) branched within 12 monophyletic clusters (aLRT > 0.75) of variable size (2–30 individuals) that mostly comprise sequences from the Sate of Goiás (63–100%) (Supplementary Figure 1). Most clusters of small sizes (2–6 individuals) only comprise sequences from young MSM analyzed in this study, while clusters of large size (8–30 individuals) comprise HIV-1 sequences from both males and females from Goiás state that were sampled in this and in previous studies (Table 4). The ML analysis of subtype F1 revealed that most sequences from MSM individuals from Goiânia here recovered (*n* = 14, 88%) branched in a single monophyletic cluster (aLRT = 1), called F1-I (Supplementary Figure 2). Besides the 14 sequences from this study, the F1-I transmission cluster also contains three additional sequences: one from a 26-year-old bisexual male sampled in Goiás state in 2007, one from a 28-year-old MSM sampled in Maranhão state (Northeast region) in 2012, and another sequence from a 41-year-old male (unknown transmission group) sampled in Amapá state (North region) in 2014.

**TABLE 4:** Subtype B and F1 transmission clusters comprising MSM individuals from Goiânia, Central-Western Brazil.

**Cluster**	***N***	**Gender/exposure category**	**GO**	**GO-MSM**	**Recruitment networks**	**T_*MRCA*_ (95% HPD)**
B-I	30	Male-MSM (23%), -Het (27%), -ND (10%); female (33%); unknown (7%)	21 (70%)	6 (20%)	B (*n* = 2), C (*n* = 4)	1982 (1975–1989)
B-II	21	Male-MSM (29%), -Het (14%), -ND (9%); female (43%); unknown (5%)	20 (95%)	3 (14%)	A (*n* = 1), C (*n* = 2)	1982 (1975–1989)
B-III	8	Male-MSM (50%), -ND (13%); female (37%)	8 (100%)	2 (25%)	E (*n* = 2)	1989 (1981–1994)
B-IV	8	Male-MSM (88%); unknown (12%)	5 (63%)	4 (50%)	C (*n* = 2), E (*n* = 2)	1984 (1976–1991)
B-V	6	Male-MSM (100%)	6 (100%)	6 (100%)	B (*n* = 1), C (*n* = 2), D (*n* = 1), E (*n* = 2)	2000 (1993–2006)
B-VI	3	Male-MSM (100%)	3 (100%)	3 (100%)	C (*n* = 1), D (*n* = 1), E (*n* = 1)	2008 (2004–2011)
B-VII	3	Male-MSM (66%); unknown (34%)	2 (66%)	2 (66%)	C (*n* = 1), E (*n* = 1)	1985 (1976–1992)
B-VIII	2	Male-MSM (50%); female (50%)	2 (100%)	1 (50%)	D (*n* = 1)	1999 (1992–2005)
B-IX	2	Male-MSM (100%)	2 (100%)	2 (100%)	C (*n* = 1), E (*n* = 1)	1991 (1982–2000)
B-X	2	Male-MSM (100%)	2 (100%)	2 (100%)	A (*n* = 1), E (*n* = 1)	2006 (2000–2011)
B-XI	2	Male-MSM (100%)	2 (100%)	2 (100%)	C (*n* = 1), E (*n* = 1)	2012 (2010–2014)
B-XII	2	Male-MSM (50%); female (50%)	2 (100%)	1 (50%)	C (*n* = 1)	1993 (1986–1998)
F1-I	17	Male-MSM (94%), -ND (6%)	15 (88%)	14 (82%)	A (*n* = 7), B (*n* = 1), C (*n* = 1), D (*n* = 2), E (*n* = 3)	2005 (2002–2006)

*MSM, men who have sex with men; N, number of isolates; GO, Goiás state; Het, heterosexual; ND, not determined;* T*_MRCA,_ time of the most recent common ancestor. Recruitment networks A–E as described in Figure 1.*

**FIGURE 4:**
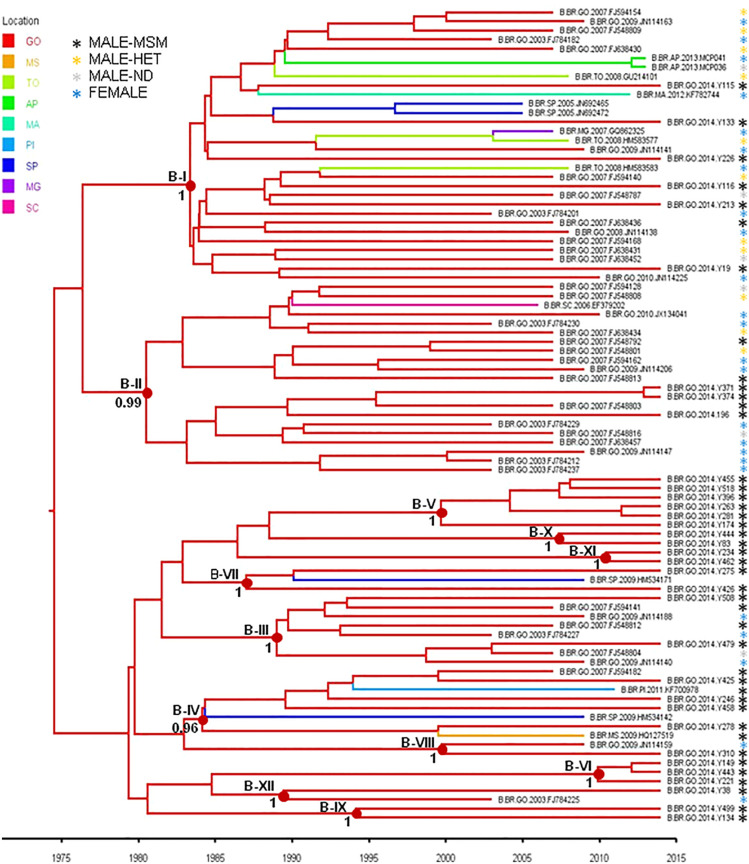
Time-scaled Bayesian Markov Chain Monte Carlo (MCMC) tree of HIV-1-subtype B *pol* sequences from the 12 transmission clusters among MSM from Central-Western Brazil. Branches are colored according to the most probable location state of their descendent nodes as indicated in the legend at the upper left side. Sequences are marked with an asterisk colored according to the risk group. Internal circles indicate the positions of the ancestral nodes of each subtype B cluster. Numbers at ancestral nodes indicate the clade support (*PP*) of subtype B transmission cluster. Branch lengths are depicted in units of time (years). The tree was automatically rooted under the assumption of a relaxed molecular clock. Location states: Goiás (GO), Mato Grosso do Sul (MS), Tocantins (TO), Amapá (AP), Maranhão (MA), Piauí (PI), São Paulo (SP), Minas Gerais (MG), Santa Catarina (SC).

**FIGURE 5:**
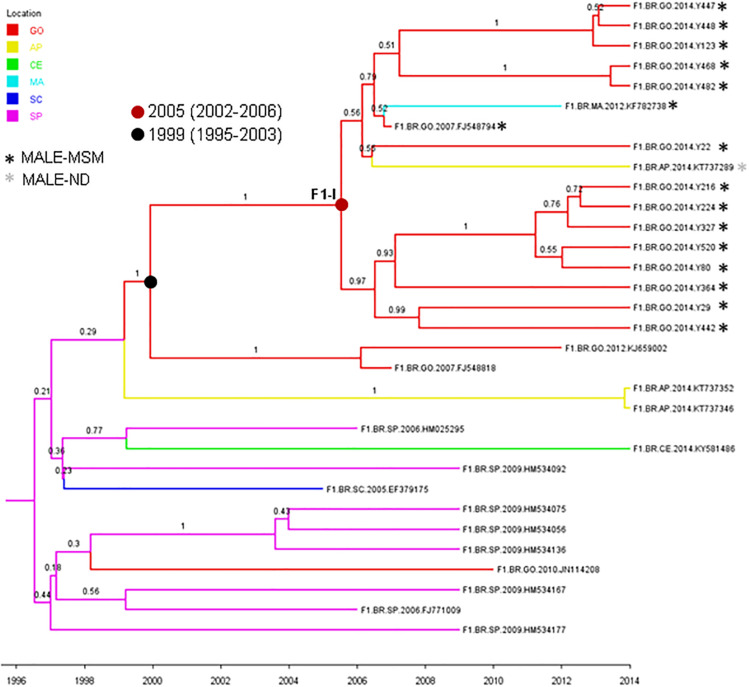
Time-scaled Bayesian Markov Chain Monte Carlo (MCMC) tree of 14 HIV-1-subtype F1 *pol* sequences from MSM from Central-Western Brazil and related sequences. Branches are colored according to the most probable location state of their descendent nodes as indicated in the legend at the upper left side. Sequences are marked with an asterisk colored according to the risk group. Internal circles indicate the positions of the ancestral of nodes of the major F1-I MSM cluster (red circle) and of basal related sequences from Goiás state (black circle). The T_*MRCA*_ of those clusters are indicated. Numbers at ancestral nodes indicate the clade support (*PP*). Branch lengths are depicted in units of time (years). The tree was automatically rooted under the assumption of a relaxed molecular clock. Location states: Goiás (GO), yellow to Amapá (AP), green to Ceará (CE), light blue to Maranhão (MA), dark blue to Santa Catarina (SC), and rose to São Paulo (SP).

To estimate the time and location of the ancestral strains that originated the subtype B and F1 transmission clusters described above, we used a Bayesian phylogeographic approach. The 12 subtype B clusters were combined in a single analysis, while the subtype F1 cluster was combined with 15 basal Brazilian sequences that branched together with the subtype F1 cluster with high support (aLRT = 0.95) in the ML phylogenetic tree. The overall topology of the ML and Bayesian trees was fully consistent (Figures 4, 5). Bayesian phylogeographic reconstructions support that all subtype B clusters analyzed most probably arose in the state of Goiás [*Posterior State Probability* (PSP) = 1] during the 1980s (clusters B-I, B-II, B-III, B-IV, and B-VII), 1990s (B-VIII, B-IX, and B-XII), 2000s (B-V, B-VI, and B-X), and 2010s (B-XI) (Figure 4 and Table 4). In the two major clusters B-I and B-II, sequences from MSM and females from Goiás state were highly intermixed among each other (Figure 4). The T_*MRCA*_ of the MSM F1-I cluster that disseminated in Goiás was estimated around the mid 2000s (Figure 5 and Table 4). Several F1 sequences from various Brazilian states branched basal to the F1 clade, but the two closest ones were from the Goiás state, suggesting that this F1 lineage most probably arose in this Brazilian state (*PSP* = 1) around the late 1990s and has been transmitted locally since then.

The great majority of 78 HIV-1 sequences obtained were from ART-naïve patients (*n* = 75), and 17.3% presented TDR (*n* = 13; CI 95%: 9.6–27.8). As shown in Table 5, most individuals with TDR were young adults (aged 18–26 years) and infected with subtype B (84.6%, *n* = 11), one with subtype F1 and one with a BF1 recombinant. Non-nucleoside reverse transcriptase inhibitor (NNRTI) mutations predominated (14.7%, 11 out of 75), followed by nucleoside reverse transcriptase inhibitor (NRTI) mutations (5.3%, 4 out of 75) and protease inhibitor (PI) mutations (1.3%, 1 out of 75). Of these, three sequences had dual-class resistance mutations to NRTI and NNRTI. Overall, six (8%) isolates had singleton mutations and seven (9.4%) presented multiple mutations. The oldest patient in this group (46 years) had multiple resistance mutations in his viral sequence (BRGO Y-462): four NRTI resistance mutations and five NNRTI mutations. K103N (NNRTI) was the most prevalent resistance mutation detected (6.7%, 5 out of 75). Of 78 samples sequenced, three were from individuals taking HIV-1 antiretroviral treatment and two of them (aged 45 and 49 years) presented DRM. These isolates (subtype B) had singleton mutations: D67N to NRTI (isolate BRGO Y115) and V106I to NNRTI (isolate BRGO Y508).

**TABLE 5:** Resistance mutations and amino acid substitutions in protease and reverse transcriptase regions in 13 HIV-1 isolates from ART-naïve MSM with transmitted drug resistance from Goiânia, Central-Western Brazil.

**MSM**	**Age (years)**	**HIV subtype PR/RT**	**Resistance mutations**			**ARV resistance profile^*a*^**		
			**PI major**	**NRTI**	**NNRTI**	**Low**	**Intermediate**	**High**
BRGO Y-19	41	BB	–	–	K101E	DOR, EFV, ETR		
BRGO Y-77	21	BB	–	–	E138G,V179E	EFV, ETR, NVP, RPV		
BRGO Y-96	19	BB	–	–	E138A, G190R	ETR, RPV		
BRGO Y-119	18	BB	–	–	K103N, P225H			EFV, NVP
BRGO Y-134	26	BB	–	M41L, L210W T215D	–		ABC, DDI, TDF	AZT, d4T
BRGO Y-137	24	BB	–	–	V179D	EFV, ETR, NVP, RPV		
BRGO Y-192	22	BB	M46L	–	–	ATV, LPV		
BRGO Y-226	21	BB	–	–	V179D	EFV, ETR, NVP, RPV		
BRGO Y-234	45	BB	–	D67N, K70R, M184V, K219Q	K101H, K103N, V108I, F227L	TDF, ETR, RPV	AZT	ABC, FTC, 3TC, EFV, NVP
BRGO Y-396	25	BB	–	–	K103N			EFV, NVP
BRGO Y-442	22	FF	–	–	K103N			EFV, NVP
BRGO Y-457	24	FB	–	M184I	G190R	ABC		FTC, 3TC
BRGO Y-462	46	BB	–	D67N, K70R, M184V, T215F, K219Q	K101H, K103N, V108I, F227L	ETR, RPV	TDF	ABC, AZT, FTC, 3TC, EFV, NVP

*HIV, human immunodeficiency virus; MSM, men who have sex with men; PR, protease region; RT, reverse transcriptase region; ART, antiretroviral therapy; ARV, antiretroviral; PI, protease inhibitor; NRTI, nucleoside reverse transcriptase inhibitors; NNRTI, non-nucleoside reverse transcriptase inhibitors, 3TC, lamivudine; ABC, abacavir; ATV, atazanavir; AZT, zidovudine; d4T, stavudine; DDI, didanosine; DOR, doravirine; EFV, efavirenz; ETR, etravirine; FTC, emtricitabine; LPV, lopinavir; NVP, nevirapine; RPV, rilpivirine; TDF, tenofovir. ^*a*^ARV resistance profile: low, intermediate and high, according to the Stanford Program HIVdb Genotypic Resistance Interpretation Algorithm.*

## Discussion

To our knowledge, this is the first study to investigate the prevalence, associated factors, and molecular characteristics of HIV-1 isolates in a population of MSM in Central-Western Brazil, using the RDS as the recruitment method. The combination of phylogenetic and phylogeographic analyses of HIV-1 *pol* sequences with epidemiological data here presented provides evidence concerning the virus dissemination in this key population.

The sociodemographic characteristics of the studied population were similar to those reported in other Brazilian MSM populations (Kerr et al., 2018), such as mostly young, self-reported as brown, single, and with 10–12 years of formal education. However, these characteristics can also be influenced by the RDS sampling method, which depends on a connected social network, and like other sampling methods for key populations, these are tradeoffs (Harling and Tsai, 2019). The weighted prevalence of HIV-1 estimated in this study (17.6%; 95% CI: 12.6–23.5) is almost 84 times higher than that found among local blood donors (0.21%; 95% CI: 0.19–0.24) (Pessoni et al., 2019), but similar to that observed in a recent multicentric study of Brazilian MSM (18.4%; CI 95%: 15.4–21.7) (Kerr et al., 2018). Regarding studies conducted in other countries with a low endemicity for HIV-1 infection, similar prevalence rates were also reported in MSM in Chile (17.6%; 95% CI: 9.6–26.0) (Stuardo Ávila et al., 2020), Mexico (20.2%; 95% CI: 12.5–29.1) (Pitpitan et al., 2015), and Amsterdam (19.0%; 95% CI: 17.6–20.4) (Achterbergh et al., 2020), indicating that MSM remain a highly vulnerable population for HIV-1 acquisition.

As reported elsewhere (Guimarães et al., 2018; Rocha et al., 2020), high frequencies of risky sexual behaviors were observed among MSM studied. Indeed, receptive anal intercourse and history of STI were factors independently associated with HIV-1 exposure, which are consistent with data found in other MSM populations (Carneiro et al., 2003; Guimarães et al., 2013). Mucosal lesions caused by anal intercourse have been associated with transmission of HIV-1 and other STI; especially by unprotect anal intercourse (Arien et al., 2011). Here, we found that half of the MSM investigated reported neglecting condom use during sexual intercourse. Of note, Brazilian data from 2009 to 2016 showed that unprotected receptive anal sex among young MSM (<25 years old) increased by 24% (Guimarães et al., 2018). In this study, sex with drug user partner was also independently associated with HIV-1 infection. This association has been previously reported to increase HIV-1 exposure (Barcelos et al., 2003). Besides that, multivariate analysis showed that HIV-1 infection was significantly associated with self-reported black skin color, which has been a marker of social vulnerability. In fact, most of black MSM were in lower tiers of economic classes (D, E) and also reported high-risk sexual behavior, even though they had attended high school (data not shown). Of note, sexual education at Brazilian schools is still a taboo and may impact HIV/AIDS knowledge and preventive measures (Guimarães et al., 2018).

The predominance of subtype B and the presence of subtypes F1 and C and BF1 recombinants corroborate previous studies which have indicated that these are the most prevalent HIV-1 variants in Goiás and other Brazilian states (Arruda et al., 2018; Reis et al., 2019). These findings were also concordant with those reported in other Brazilian MSM populations (Bermúdez-Aza et al., 2011; Tupinambás et al., 2013). The detection of subtype C strains in the Brazilian Central-West region was already described (Arruda et al., 2018; Reis et al., 2019), suggesting the continuous dissemination of this viral variant away from its epicenter in the South region. Here, BF1 recombinants were found in a rate of 10.3%, corroborating the frequency of 9% reported by Reis et al. (2019) in a recent study that summarizes data from previous studies conducted in Goiás and highlights the importance of BF1 recombinants in the HIV/AIDS epidemic from Central-Western Brazil. Among the eight BF1 recombinants from MSM studied, six showed distinct recombination profiles, demonstrating the high genetic diversity of these isolates. In the phylogenetic tree of BF1 recombinants, two sequences (BRGO Y13 and BRGO Y65 from MSM sex workers) formed a cluster that branched with CRF47_BF, which was originated in North-West of Spain (Fernández-García et al., 2010). Therefore, further studies of full-length or near full-length genome sequences of HIV BF1 isolates here obtained are important to clarify the phylogenetic relationships between these hybrids and the CRFs already described and also to identify possible new CRFs_BF1.

In this study, a higher frequency of subtype F1 (20.5%) was observed in comparison to those reported in other populations of the same region (Reis et al., 2019), indicating that subtype F1 has an increased circulation in MSM from Goiânia. Similarly, high rates of this subtype were found among MSM population from Belo Horizonte in the Southeastern region (Tupinambás et al., 2013). Phylogenetic analysis showed that most (88%) subtype F1 isolates from MSM in Goiânia were part of a single transmission cluster (here called F1-I) that probably circulates locally since the late 1990s and entered in the MSM population around the mid-2000s. Our phylogeographic analysis indicates that this F1-I cluster was not restricted to Goiás but was also disseminated to men living in Amapá (North region) and Maranhão (Northeast region). We observed that half of the sequences (7 out of 14) from Goiânia that branched within cluster F1-I was from MSM that belonged to the same recruitment network (A) and the other seven belonged to the remaining recruitment networks (B, C, D, and E), reinforcing their spreading among MSM from Goiânia, thus influencing local dynamic of HIV-1 infection in this population. Additionally, the frequencies of black skin color self-report (*p* < 0.001), receptive anal intercourse (*p* < 0.001), and sex with STI partner (*p* = 0.015) were significantly higher in MSM infected with F1 sequences that formed the transmission cluster compared to those infected with non-F1 isolates (data not shown).

A great proportion (67%) of subtype B sequences of MSM from Goiânia was distributed across several clusters of variable size (2–30 individuals) that most probably arose in the state of Goiás during the 1980s (clusters B-I, B-II, B-III, B-IV, and B-VII), 1990s (B-VIII, B-IX, and B-XII), 2000s (B-V, B-VI, and B-X), and 2010s (B-XI). The small subtype B clusters (B-V to B-XII) were mostly restricted to MSM from Goiás. The largest subtype B clusters, by contrast, circulate in both males and females and were also disseminated to the Southeastern (B-I and B-IV), Northeastern (B-I and B-IV), Southern (B-II), and Northern (B-I) regions. We observed that 33–100% of the sequences from Goiânia that grouped into B-I, B-II, B-III, B-IV, and B-V clusters were from MSM that belonged to the same recruitment network. On the other hand, we also noted that all recruitment networks (A–E) presented MSM that were infected with isolates belonging to different B clusters (Table 4). In addition, no significant association was detected between MSM sociodemographic or behavioral characteristics and subtype B clusters (data not shown). Taken together, these findings indicate the expansion of long-standing local transmission networks of subtype B, as well as its dissemination between different Brazilian regions, including groups of different exposure categories.

According to the WHO classification criteria, a high TDR prevalence of 17.3% was found among MSM (Bennett et al., 2009) which is higher than the overall TDR rate (9.5%) determined in a recent nationwide study in Brazil, in which the lowest rate (6.8%) was observed in Central-Western Region (Arruda et al., 2018). Nevertheless, relative to other Brazilian MSM populations, the prevalence found in this study was lower than the overall TDR rate reported in nine Brazilian cities (21.4%) (Bermúdez-Aza et al., 2011), but was, however, higher than that shown in the city of Belo Horizonte, Southeast region (14.1%) (Tupinambás et al., 2013).

From the public health perspective, we also observed that five out 13 clusters (38.5%; B-I, -V, -IX, -XI, and F-I) contained samples with TDR. In one of them (cluster B-XI), sequences from MSM Y-234 and Y-462 shared multiple NRTI (D67N, K70R, M184V, and K219Q) and NNRTI (K101H, K103N, V108I, and F227L) mutations, indicating the possibility of transmission of multidrug resistance between these individuals. These findings demonstrate the existence of transmission clusters associated with drug resistance indicating the need to intensify HIV-1 preventive interventions in addition to continued surveillance of TDR among MSM for mitigating drug resistance and its transmission in this key population.

It is noteworthy that in our study group 9.4% of HIV-1 isolates had multiple mutations that may impact the response to ARV treatment. As reported elsewhere (Arruda et al., 2018), NNRTI mutations predominated in our study and the 14.7% prevalence rate here found was higher than those reported in a Brazilian nationwide study (5.8%; ranging from 4.5% in Central-West and Northeast to 7.0% in South) (Arruda et al., 2018). The most frequent NNRTI mutation detected, K103N (6.7%) was also observed in high rates in other studies conducted in Brazil (Bermúdez-Aza et al., 2011; Arruda et al., 2018; Crispim et al., 2019; Tanaka et al., 2019). It is a non-polymorphic mutation that causes high-level resistance to nevirapine (NVP) and efavirenz (EFV). The latter was in the first-line ARV regimen until 2016 when it was replaced by dolutegravir (DTG) (Brasil. Ministério da Saúde. Secretaria de Vigilância em Saúde. Departamento de Vigilância Prevenção e Controle das Ist, do HIV/AIDS e das Hepatites Virais – DIAHV, 2018a). Thus, the high prevalence of mutations to NNRTI, such as K103N, probably reflects a selective pressure exerted by EFV, selecting resistant mutants that can be transmitted and potentially impact the results of therapy. NNRTI mutations were followed by NRTI (5.3%). Of note, tenofovir (TDF) and lamivudine (3TC) were in the first-line ARV regimen for adults since 2013 in Brazil (Brasil. Ministério da Saúde. Secretaria de Vigilância em Saúde. Departamento de Vigilância Prevenção e Controle das Ist, do HIV/AIDS e das Hepatites Virais – DIAHV, 2013, 2018a). In our study, four MSM HIV-1 isolates (BRGO Y-134 Y-234, Y-457, and Y-462) presented mutations to NRTI that may influence the use of 3TC (M184V/I mutations) and TDF (M41L, L210W, T215D, T215F, D67N, and K70R mutations). In addition to resistance to TDF, these isolates also presented resistance mutation to emtricitabine (FTC) (M184V/I), which could impact the use of pre-exposure prophylaxis (PrEP) introduced for this population in 2017 in Brazil (Brasil. Ministério da Saúde. Secretaria de Vigilância em Saúde. Departamento de Vigilância Prevenção e Controle das Ist, do HIV/AIDS e das Hepatites Virais – DIAHV, 2018b). On the other hand, only the M46L PI resistance mutation was detected, which is associated with reduced susceptibility to atazanavir (ATV) and lopinavir (LPV). The low prevalence of PI mutations found (1.3%) is in accordance with other national studies (Arruda et al., 2018; Crispim et al., 2019; Tanaka et al., 2019).

This study has some limitations. This was a cross-sectional study, and behavioral findings are based on self-reports, which can overestimate or underestimate the chances of HIV-1 positivity. Although some limitations of the RDS have been well reviewed (Abdesselam et al., 2020), it is commonly used and will continue to be used for obtaining epidemiological data of hard-to-reach populations, such as MSM, until better methods are available. Concerning the molecular analysis of HIV-1, it was based on sequence analysis of the *pol* gene, including the entire PR and partial RT regions. Therefore, further investigations using full-length or near-full-length genomic analysis are necessary to better characterize mainly the subtype F1 and BF1 recombinant HIV-1 isolates.

## Conclusion

This study demonstrates a high prevalence of HIV-1 among MSM in the city of Goiânia, Central-Western Brazil, which was associated with risky sexual behaviors. Our results highlight the high genetic diversity of HIV-1 among MSM, which is driven by the local expansion of different subtype B and F1 strains that are probably circulating locally since the 1980s and late 1990s, respectively, and belonging to transmission clusters. The findings of high TDR rate and the description of the resistance mutations support the need of continued surveillance HIV-1 drug resistance in this key population.

## Data Availability Statement

The datasets generated for this study can be found in online repositories. The names of the repository/repositories and accession number(s) can be found in the article/Supplementary Material.

## Ethics Statement

The studies involving human participants were reviewed and approved by the Ethics Committee of the Federal University of Goiás (No. 497374). The participants provided their written informed consent to participate in this study.

## Author Contributions

RM and MS conceived the study and obtained funding. ÁS, NF, ST, MM, MC, and RM collected and analyzed the epidemiological data. ÁS and MR conducted the sequencing and submitted sequence information to GenBank. ÁS, MR, and GB performed the bioinformatics analyses. ÁS, TM, GB, RM, and MS wrote the first draft of the manuscript. All authors read and approved the submitted version.

## Conflict of Interest

The authors declare that the research was conducted in the absence of any commercial or financial relationships that could be construed as a potential conflict of interest.

## Footnotes

1 https://hiv.lanl.gov

2 http://hivdb.stanford.edu

3 http://rds-analysis-tool.software.informer.com/versions/

4 http://www.analytictech.com/downloadnd.htm

## Supplementary Material

Supplementary Figure 1 Maximum likelihood (ML) phylogenetic tree of HIV-1 subtype B *pol* sequences (∼1,000 nt) from MSM individuals from Goiás described in the present study (red branches), combined with subtype B sequences from Goiás (blue branches) and other Brazilian states (black branches) that were available at the Los Alamos HIV Sequence Database. Shaded boxes highlight the position of the subtype B monophyletic clusters (*a*LRT > 0.75) containing sequences from MSM from Goiás here described. Tree was rooted on midpoint. The branch lengths are drawn to scale with the bar at the bottom indicating nucleotide substitutions per site.

Supplementary Figure 2 Maximum likelihood phylogenetic tree of HIV-1 subtype F-1 *pol* sequences (∼1,000 nt) from MSM individuals from Goiás described in the present study (red branches), combined with subtype F1 sequences from Goiás (blue branches) and other Brazilian states (black branches) that were available at the Los Alamos HIV Sequence Database. Shaded boxes highlight the position of the subtype F1 monophyletic cluster (*a*LRT = 0.95) containing most sequences from MSM from Goiás here described. Tree was rooted on midpoint. The branch lengths are drawn to scale with the bar at the bottom indicating nucleotide substitutions per site.
