# Supplementary figures and images for: Epidemiological and Molecular Characteristics of HIV-1 Infection in a Sample of Men Who Have Sex With Men in Brazil: Phylogeography of Major Subtype B and F1 Transmission Clusters
Source: Front Microbiol. 2020 Nov 27;11:589937. doi: 10.3389/fmicb.2020.589937 (PMC7732656; doi:10.3389/fmicb.2020.589937)

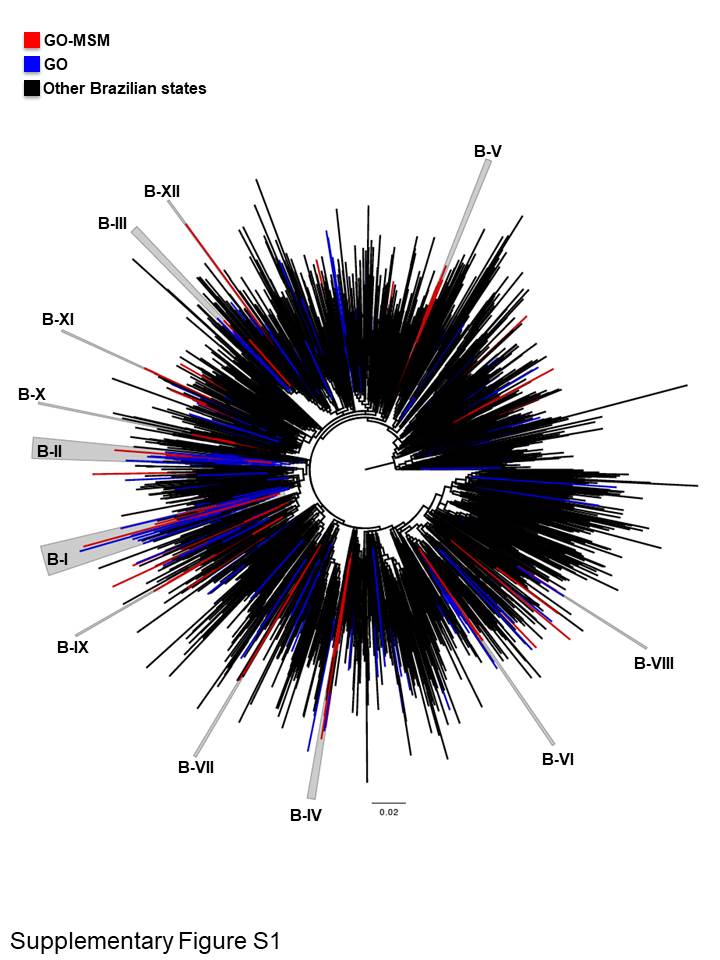

Supplement: Supplementary Figure 1 — Maximum likelihood (ML) phylogenetic tree of HIV-1 subtype B pol sequences (∼1,000 nt) from MSM individuals from Goiás described in the present study (red branches), combined with subtype B sequences from Goiás (blue branches) and other Brazilian states (black branches) that were available at the Los Alamos HIV Sequence Database. Shaded boxes highlight the position of the subtype B monophyletic clusters (aLRT > 0.75) containing sequences from MSM from Goiás here described. Tree was rooted on midpoint. The branch lengths are drawn to scale with the bar at the bottom indicating nucleotide substitutions per site. [file Image_1.jpg]

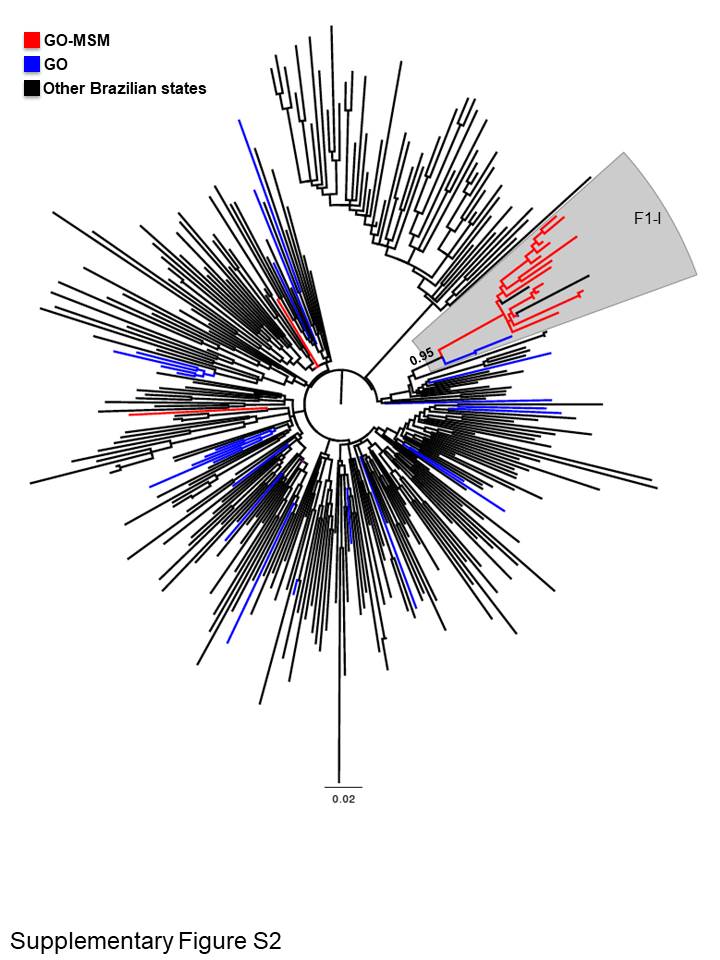

Supplement: Supplementary Figure 2 — Maximum likelihood phylogenetic tree of HIV-1 subtype F-1 pol sequences (∼1,000 nt) from MSM individuals from Goiás described in the present study (red branches), combined with subtype F1 sequences from Goiás (blue branches) and other Brazilian states (black branches) that were available at the Los Alamos HIV Sequence Database. Shaded boxes highlight the position of the subtype F1 monophyletic cluster (aLRT = 0.95) containing most sequences from MSM from Goiás here described. Tree was rooted on midpoint. The branch lengths are drawn to scale with the bar at the bottom indicating nucleotide substitutions per site. [file Image_2.jpg]
